# MRI texture features may predict differentiation and nodal stage of cervical cancer: a pilot study

**DOI:** 10.1177/2058460117729574

**Published:** 2017-10-17

**Authors:** Anton S Becker, Soleen Ghafoor, Magda Marcon, Jose A Perucho, Moritz C Wurnig, Matthias W Wagner, Pek-Lan Khong, Elaine YP Lee, Andreas Boss

**Affiliations:** 1 Department of Diagnostic and Interventional Radiology, University Hospital of Zurich, Zurich, Switzerland; 2 Department of Diagnostic Radiology, The University of Hong Kong, Hong Kong, PR China

**Keywords:** Cervical cancer, texture features, texture analysis, apparent diffusion coefficient (ADC), diffusion-weighted imaging (DWI)

## Abstract

**Background:**

Texture analysis in oncological magnetic resonance imaging (MRI) may yield surrogate markers for tumor differentiation and staging, both of which are important factors in the treatment planning for cervical cancer.

**Purpose:**

To identify texture features which may predict tumor differentiation and nodal status in diffusion-weighted imaging (DWI) of cervical carcinoma

**Material and Methods:**

Twenty-three patients were enrolled in this prospective, institutional review board (IRB)-approved study. Pelvic MRI was performed at 3-T including a DWI echo-planar sequence with b-values 40, 300, and 800 s/mm^2^. Apparent diffusion coefficient (ADC) maps were used for region of interest (ROI)-based texture analysis (32 texture features) of tumor, muscle, and fat based on histogram and gray-level matrices (GLM). All features confounded by the ROI size (linear model) were excluded. The remaining features were examined for correlations with histological differentiation (Spearman) and nodal status (Kruskal–Wallis). Hierarchical cluster analysis was used to identify correlations between features. A *P* value < 0.05 was considered statistically significant.

**Results:**

Mean age was 55 years (range = 37–78 years). Biopsy revealed two well-differentiated, eight moderately differentiated, two moderately to poorly differentiated tumors, and five poorly differentiated tumors. Six tumors could not be graded. Lymph nodes were involved in 11 patients. Three GLM features correlated with the differentiation: LRHGE (ϱ = 0.53, *P* = 0.03), ZP (ϱ = –0.49, *P* < 0.05), and SZE (ϱ = –0.51, *P* = 0.04). Two histogram features, skewness (0.65 vs. 1.08, *P* = 0.04) and kurtosis (0.53 vs. 1.67, *P* = 0.02), were higher in patients with positive nodal status. Cluster analysis revealed several co-correlations.

**Conclusion:**

We identified potentially predictive GLM features for histological tumor differentiation and histogram features for nodal cancer stage.

## Introduction

Invasive carcinoma of the uterine cervix (cervical cancer) is the fourth most common cause of death by cancer in the Western world and the second most common cause of death in the developing world (1). In Europe, recent widespread adaptations of human papillomavirus (HPV) vaccination and HPV-screening programs have greatly contributed to decrease the incidence of invasive cervical cancer (2). For affected patients, accurate staging and tumor grading has led to more efficient therapies with better outcomes and reduced side effects: while patients with low-grade and locally confined tumors (Fédération de Gynécologie et d’Obstétrique [FIGO] stages IB1 and IIA1) may be amenable to surgical resection alone, advanced and poorly differentiated tumors require combined radio-chemotherapy (3). Magnetic resonance imaging (MRI) offers individualized assessment for affected patients, since it not only provides detailed anatomical information but also extracts functional information, e.g. via dynamic contrast-enhanced or diffusion-weighted imaging (DWI).

Texture analysis is a mathematical–statistical procedure to extract objective and quantitative parameters (texture features) from given images (4). It was proposed and established in the second half of the last century, but only in recent years has it been used to characterize and measure tissue heterogeneity in medical images. Texture analysis may detect subtle, sub-resolution changes in tumor morphology—changes otherwise not visible to the radiologist’s eye.

From any medical image, which is essentially a matrix of gray levels, a variable number of features can be extracted and mined for associations with clinical data and other biomarkers. This methodology has recently been coined as the “radiomics” approach. In their landmark paper, Aerts et al. (4) have found many features which could be used as surrogate markers for underlying gene expression patterns as well as relevant clinical outcome data. Furthermore, some texture features correlate consistently with biological tumor traits across different cancers and studies: for example, malignant adnexal masses exhibit high intratumoral entropy and benign masses exhibit low intratumoral entropy (5), whereas in prostate carcinoma, a positive correlation between entropy and the histological Gleason grade was shown (6). In both of these studies, texture analysis was performed on DWI, which conveys information about the tissue cellularity.

Since DWI has already been shown to be useful in diagnostic imaging of cervical carcinoma (7), the study of texture features may add additional diagnostic value and has yet to be examined. Thus, the purpose of this study was to identify texture features in DWI which may predict differentiation and nodal status of cervical carcinoma.

## Material and Methods

### Patients

This prospective clinical study was approved by the institutional review board (IRB). Oral and written consent was obtained from all patients. Twenty-three female patients (mean age = 55 years; age range = 37–78 years) with biopsy-proven cervical carcinoma in the years 2014–2015 were enrolled. None of the patients had received prior treatment. Clinical FIGO stage was obtained from the electronic patient record.

### Histopathological analysis

Histopathological specimens were assessed by an experienced gynecological pathologist for tumor type and differentiation (G1–G3). Additionally, all cases were reviewed at the weekly multidisciplinary tumor board together with gynecologists and radiologists.

### Imaging protocol

All examinations were performed on a clinical 3-T MRI scanner (Achieva 3.0 T TX, Philips Healthcare, Best, The Netherlands), using a 16-channel matrix torso coil. Patients had to fast 6 h before the examination. A total of 20 mg hyoscine butylbromide (Buscopan, Boehringer Ingelheim, Germany) were administered as an intramuscular injection immediately before scanning to reduce bowel peristalsis. Diffusion datasets of the pelvis were acquired with a single-shot spin-echo echo-planar imaging (SS SE EPI) sequence in axial orientation (20 slices) to include the entire cervical cancer using the following b-values: 40, 300, 800 s/mm^2^ averaged in three orthogonal directions. Sequences were acquired in free-breathing. Fat suppression was used by spectral presaturation with inversion recovery (SPIR). Apart from the DWI sequence, anatomical T1-weighted (T1W) and T2-weighted (T2W) sequences were acquired as part of the clinical scan protocol (Table 1). Apparent diffusion coefficient (ADC) maps were automatically computed from the DW images using a mono-exponential decay model. This model assumes that the increasing signal loss with increasing b-values is only attributable to molecular diffusion. Even though it ignores other effects, such as microvascular perfusion at low b-values, the ADC is the most commonly used DWI-derived parameter in the clinical routine. The ADC correlates inversely with cellularity, i.e. a lower ADC usually corresponds to an increase in cells per volume.

**Table 1.** Summary of the MRI protocol.

Sequences	T2W TSE	T2W SPAIR	T2 TSE	T2W TSE	DWI	CE T1W- THRIVE
Plane	Sagittal	Coronal	Axial	Oblique axial	Axial	3D
TR/TE (ms)	4000/80	3500/80	2800/100	2800/100	2000/54	03.01.04
Turbo factor	30	21	12	14	NA	NA
Field of view (mm)	240 × 240 | 230 × 230	402 × 300 | 220 × 220	406 × 300 | 370 × 203			
Matrix size	480 × 298 | 352 × 300	787 × 600 | 316 × 311	168 × 124 | 248 × 134			
Slice thickness (mm)	4	4	4	4	4	1.5
Intersection gap (mm)	0	0	0	0	0	0
Bandwidth (Hz/pixel)	230	186	169	162	15.3	724
NEX	2	1	1	1	2	1

### Texture analysis

**Fig. 1.**
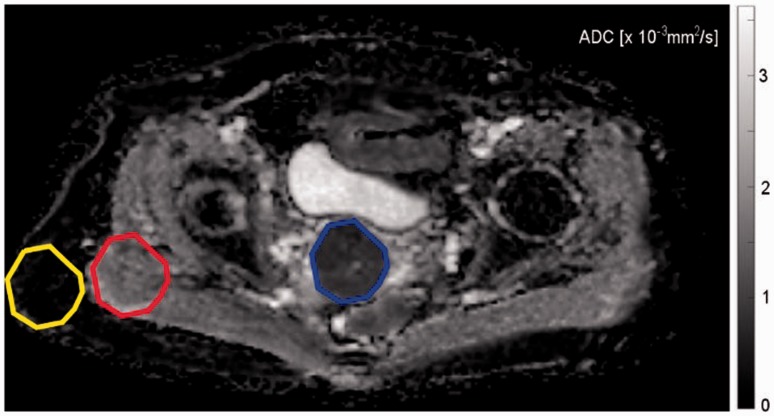
Exemplary ROI definition in a 43-year-old patient with a G2 squamous cell carcinoma of the cervix (FIGO stage IB2, T3aN1M0). The ROIs are color coded for better visibility: Blue = tumor, red = gluteal muscle, yellow = subcutaneous fat. The latter two ROIs are copies of the first one, identical in shape and size.

Texture analysis was performed in MATLAB (v2016b, The MathWorks Inc., Natick, MA, USA) with a routine based on the works of Vallières et al. (8). For the quantitative analysis, polygonal region of interest (ROI) outlines of the tumor were drawn. The ROI was then copied (to retain the same size and shape) in the subcutaneous fat and in the gluteus maximus muscle. ROI definition was performed on the slice of the ADC map depicting the largest tumor diameter in axial orientation. Absence of frank susceptibility or movement artifacts was verified for all b-values. ROI definition was performed by a PGY-3 radiology resident (ASB) and a board-certified radiologist with seven years of experience in genitourinary imaging (EYPL) in consensus. An exemplary ROI definition is shown in Fig. 1. For qualitative verification of significant differences, texture feature maps of significantly discriminative features were computed using a 5 × 5 pixel sliding patch over the whole slice, with the texture feature value of the 25 pixels assigned to the central pixel of the patch.

**Fig. 2.**
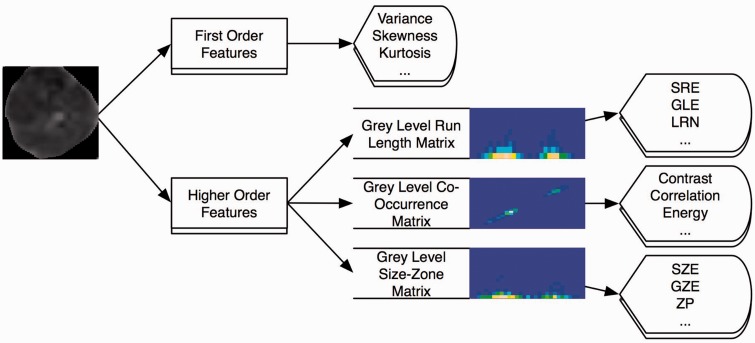
Schematic of feature extraction from an exemplary ROI. The first order features are directly derived from the histogram of the ROI content, while the higher-order features are computed from the respective gray-level matrices.

ROI contents were normalized between the mean and three standard deviations (μ ± 3 σ). This procedure minimizes intra- and interscanner effects in MRI texture analysis (9,10). Afterwards, 32 texture features were computed (Table 2). The four first order features were derived from the histogram, whereas the 28 higher order features were obtained from the gray-level co-occurrence matrix (GLCM), the gray level run length matrix (GLRLM), or the gray-level size-zone matrix (GLSZM). Fig. 2 illustrates this procedure. In comparison to first order features, where a lot of the spatial information was lost through the transformation of gray levels into counts in the histogram, GLCM, GLRLM, and GLSZM features contain more information about the distribution of gray values since they account for the location of each voxel with regard to the neighboring voxels. The four first order features describe various properties of the histogram: Variance is defined as the spread of values around the mean, kurtosis is the “peakedness” or “flatness” of the histogram and skewness is a measure of asymmetry. Lastly, entropy is a measure of uncertainty in the image voxels. Though some of the higher order features’ names sound “intuitive,” such as “contrast” or “homogeneity,” none resemble an intuitive pattern (11) (intuitive in this context meaning readily distinguishable by the human reader). The mathematical definition of each feature is beyond the scope of this paper but can be found in the works of Dasarathy and Holder for the GLCM (12), Mary M. Galloway for the GLRLM (13), and Thibault for the GLSZM (14).

**Table 2.** Texture features and abbreviations.

Histogram-derived	GLCM	GLRLM	GLSZM
Variance	Contrast	Short run emphasis (SRE)	Small zone emphasis (SZE)
Skewness	Correlation	Long run emphasis (LRE)	Large zone emphasis (LZE)
Kurtosis	Energy	Gray-level non-uniformity (GLN)	Gray-level non-uniformity (GLN)
Entropy	Homogeneity	Run length non-uniformity (RLN)	Zone-size non-uniformity (ZSN)
		Run percentage (RP)	Zone percentage (ZP)
		Low gray-level run emphasis (LGRE)	Low gray-level zone emphasis (LGZE)
		High gray-level run emphasis (HGRE)	High gray-level zone emphasis (HGZE)
		Short run low gray-level emphasis (SRLGE)	Small zone low gray-level emphasis (SZLGE)
		Short run high gray-level emphasis (SRHGE)	Small zone high gray-level emphasis (SZHGE)
		Long run low gray-level emphasis (LRLGE)	Large zone low gray-level emphasis (LZLGE)
		Long run high gray-level emphasis (LRHGE)	Large zone high gray-level emphasis (LZHGE)
			Gray-level variance (GLV)
			Zone size variance (ZSV)
			

GLCM, gray-level co-occurrence matrix; GLRLM, gray-level run-length matrix; GLSZM, gray-level size-zone matrix.

### Statistical analysis

Statistical analysis was performed using R v. 3.3.1. (R Foundation for Statistical Computing, Vienna, Austria). Continuous data were expressed as mean ± standard deviation (SD) if normal distribution could be assumed or otherwise as median and interquartile range (IQR). Categorical data were given in absolute counts. In a first step, a one-way analysis of variance (ANOVA) of a linear model was performed in all tissues to assess the influence of the ROI size on each feature. A *P* value < 0.01 in any of the tissues was considered significant for this first step. Confounded features identified in this step were excluded from further analysis. The remaining features, as well as raw ROI size, were examined for correlation with the histological grade, type, and the FIGO stage with Spearman’s rho. Differences in the groups with and without lymph node metastases were assessed with a Kruskal–Wallis test. A *P* value < 0.05 was considered statistically significant. Lastly, hierarchical cluster analysis was used to identify redundant features.

## Results

### Patients

There were eight patients with a FIGO stage I tumor, ten patients with a stage II tumor, and five patients with a stage III tumor. Only two patients had a well differentiated tumor (G1, low-grade), eight tumors were moderately differentiated (G2, intermediate grade), two were moderately to poorly differentiated (G2–3, intermediate to high grade), and five were poorly differentiated (G3, high-grade). Grading was not possible in six tumors (GX). Ten patients had a squamous cell carcinoma, four patients had an adenosquamous carcinoma, and nine patients had an adenocarcinoma. Eleven patients had cancer spread to the pelvic lymph nodes confirmed on imaging, of which three also had affected para-aortic lymph nodes. Surgical lymph node dissection was only performed in three patients negative on imaging, which was confirmed by histology. Image acquisition and post-processing were successfully completed for all patients.

### MRI tumor characteristics

Median ROI size was 219 pixels (range = 50–1809 pixels), median tumor volume measured on the sagittal T2 slices was 46.5 mL (range = 0.3–335.4 mL). Correlation between ROI size and volumetric tumor measurement was excellent (R = 0.92, *P* < 0.001). The tumors usually presented as hyperintense masses in the T2W sequences and hypointense on the ADC map compared to the surrounding cervix tissue. There were no significant artifacts in the tumor areas, nor macroscopic areas of necrosis. All tumors were clearly demarcated. Mean ADC values (×10^−3 ^mm^2^/s) were 1.01 for adenocarcinoma and 0.99 for adenosquamous and squamous cell carcinomas with no significant differences between the three types. Also, no correlation could be established between mean ADC and either tumor grade (*P* = 0.65) or FIGO stage (*P* = 0.52).

### Texture analysis

There was no correlation between raw ROI size and tumor grade (*P* > 0.5) or type (*P* > 0.6). Texture features were successfully computed for all patients and tissues. ANOVA revealed multiple features correlating with the ROI size in muscle, fat and tumor tissue shown in Table 3. In summary, the following features appeared not to be systematically influenced by the ROI size in any of the three tissues: variance, skewness, kurtosis, entropy, contrast (GLCM), correlation (GLCM), homogeneity (GLCM), SRE (GLRLM), LRE (GLRLM), RP (GLRLM), LGRE (GLRLM), HGRE (GLRLM), SRLGE (GLRLM), SRHGE (GLRLM), LRHGE (GLRLM), SZE (GLSZM), LZE (GLSZM), GLN (GLSZM), ZSN (GLSZM), ZP (GLSZM), HGZE (GLSZM), SZHGE (GLSZM), LZHGE (GLSZM), and GLV (GLSZM)

**Table 3.** One-way ANOVA (*P* values) of all tissues with the ROI size.

	Tumor	Muscle	Fat
*Variance*	*0.03*	*< 0.01*	*0.16*
Skewness	0.49	0.94	0.63
Kurtosis	0.82	0.12	0.78
Entropy	0.85	0.80	0.77
Contrast (GLCM)	0.93	0.98	0.95
*Correlation (GLCM)*	*0.01*	*< 0.01*	*0.11*
Energy (GLCM)	0.85	0.97	0.10
*Homogeneity (GLCM)*	*0.03*	*0.35*	*0.07*
SRE (GLRLM)	0.85	0.76	0.11
LRE (GLRLM)	0.95	0.84	0.24
*GLN (GLRLM)*	*< 0.01*	*< 0.01*	*< 0.01*
*RLN (GLRLM)*	*< 0.01*	*< 0.01*	*< 0.01*
RP (GLRLM)	0.97	0.80	0.12
*LGRE (GLRLM)*	*< 0.01*	*0.42*	*0.55*
HGRE (GLRLM)	0.86	0.65	0.33
SRLGE (GLRLM)	0.01	0.42	0.55
SRHGE (GLRLM)	0.86	0.54	0.24
*LRLGE (GLRLM)*	*< 0.01*	*0.43*	*0.57*
LRHGE (GLRLM)	0.75	0.74	0.24
SZE (GLSZM)	0.99	0.69	0.04
LZE (GLSZM)	1.00	0.88	0.66
GLN (GLSZM)	0.06	0.02	0.06
ZSN (GLSZM)	0.93	0.68	0.02
ZP (GLSZM)	0.98	0.77	0.06
*LGZE (GLSZM)*	*0.01*	*0.47*	*0.50*
HGZE (GLSZM)	0.95	0.41	0.26
*SZLGE (GLSZM)*	*< 0.01*	*0.47*	*0.58*
SZHGE (GLSZM)	0.95	0.39	0.36
LZLGE (GLSZM)	0.01	0.47	0.64
LZHGE (GLSZM)	0.85	0.77	0.68
GLV (GLSZM)	0.02	0.09	0.31
*ZSV (GLSZM)*	*< 0.01*	*< 0.01*	*0.05*

Confounded features are highlighted in italics (*P* < 0.01).

**Fig. 3.**
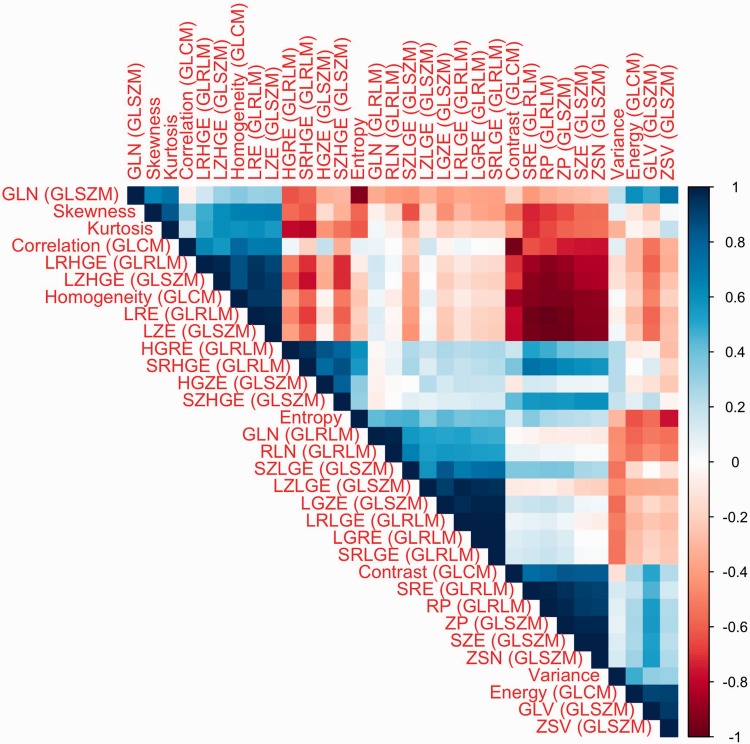
Correlation matrix of the texture features, showing significant co-correlations of several features (ordered by hierarchical clustering for better visibility): For example, ZP and SZE (6th and 7th from the bottom) correlate positively with each other, and negatively LRHGE (5th from the top). These three features may thus reflect the same (unknown) underlying biological difference.

**Fig. 4.**
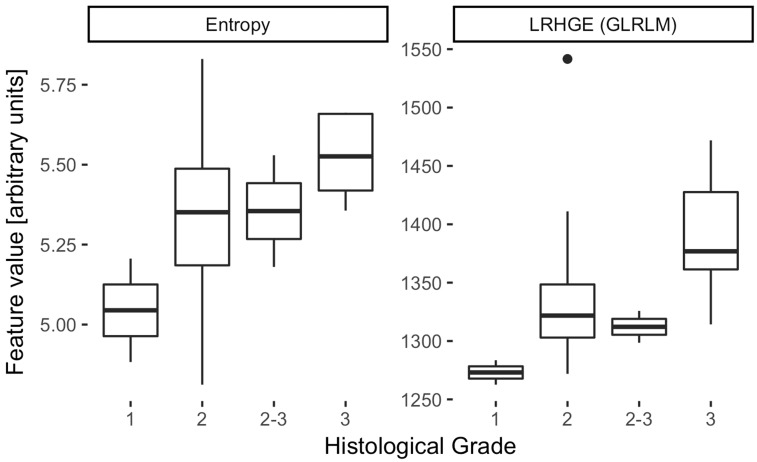
Boxplot of two texture features which independently correlate with tumor differentiation: LRHGE (ϱ = 0.53, *P* = 0.03) and entropy (ϱ = 0.49, *P* = 0.05).

**Fig. 5.**
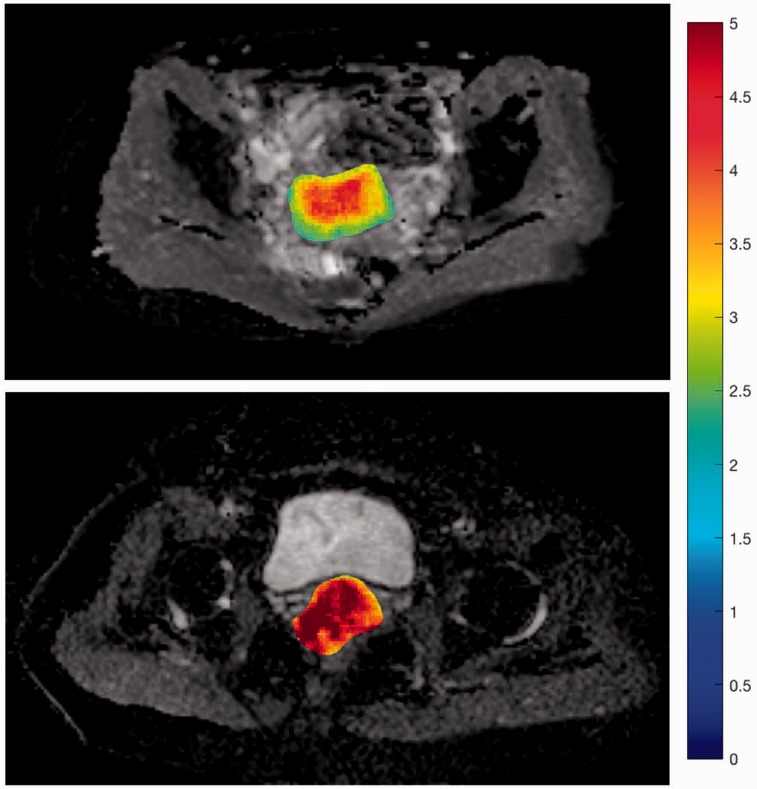
Calculated entropy-maps of two patients: 46-year-old patient with a well-differentiated (G1) squamous cell carcinoma (top) exhibiting comparably low intratumoral entropy compared to a 52-year-old patient with a poorly differentiated (G3) adenocarcinoma (bottom).

Of these 24 features, only three (LRHGE, SZE, ZP) correlated significantly with tumor differentiation (ϱ = 0.53, –0.49, and –0.51; *P* < 0.05, all *P* values given in Table 4), while none correlated with histological subtype or FIGO stage. Two first-order features (skewness and kurtosis) were significantly higher in patients with lymph node metastasis (0.65 vs. 1.08, *P* = 0.04 and 0.53 vs. 1.67, *P* = 0.02). However, in cluster analysis, the three features, ZP, SZE, and LRHGE, exhibited significant correlations with each other as did skewness and kurtosis, graphically summarized in the correlation matrix in Fig. 3. This left LRHGE and kurtosis as two independent markers for tumor differentiation and the presence of lymph node metastasis, respectively. Notably, entropy was independent of most other features and its correlation with tumor differentiation was almost statistically significant (*P* = 0.05), as depicted in the boxplot in Fig. 4 and illustrated in the “Entropy-map” in Fig. 5.

**Table 4.** Spearman correlation testing (*P* values) of the relevant features.

Feature	Tumor grade	Tumor type	FIGO stage
Variance	0.17	0.08	0.32
Skewness	0.56	0.68	0.68
Kurtosis	0.93	0.20	0.78
Entropy	0.05	0.69	0.95
Contrast (GLCM)	0.27	0.19	0.21
Correlation (GLCM)	0.07	0.17	0.28
Homogeneity (GLCM)	0.11	0.40	0.42
SRE (GLRLM)	0.08	0.78	0.57
LRE (GLRLM)	0.06	0.45	0.45
RP (GLRLM)	0.07	0.49	0.50
LGRE (GLRLM)	0.23	0.70	0.84
HGRE (GLRLM)	0.39	0.69	0.58
SRLGE (GLRLM)	0.34	0.80	0.78
SRHGE (GLRLM)	0.67	0.68	0.59
*LRHGE (GLRLM)*	*0.03*	0.34	0.41
*SZE (GLSZM)*	*0.04*	0.92	0.56
LZE (GLSZM)	0.06	0.58	0.49
GLN (GLSZM)	0.10	0.60	0.69
ZSN (GLSZM)	0.05	0.90	0.54
*ZP (GLSZM)*	*< 0.05*	0.73	0.48
HGZE (GLSZM)	0.69	0.91	0.66
SZHGE (GLSZM)	0.22	0.85	0.63
LZHGE (GLSZM)	0.09	0.43	0.31
GLV (GLSZM)	0.08	0.98	0.84

## Discussion

In the present study, we explored the diagnostic value of texture features in DWI of cervical cancer. Skewness and kurtosis differed significantly in patients with pelvic lymph node metastasis as compared to lymph node negative patients. Moreover, we found three gray-level matrix-derived features that correlate with the tumor cell differentiation grade.

First order features contain important information about underlying biological changes: in one of the few prospective studies on DWI texture features, skewness and kurtosis were found to have the potential to predict chemotherapy response in peritoneal cancer (15). Though in cervical cancer the N-stage has no influence on the clinical FIGO staging, the pelvic and para-aortic lymph node status is an important independent prognostic factor, particularly in early-stage disease (16). In addition, the presence of lymph node metastases is an important finding that will alter management for the individual patient (17). Higher skewness or kurtosis in the main tumor may thus prompt meticulous inspection of the pelvic and paraaortic lymph nodes for possible cancer spread (18). In heterogeneous tumors, areas of lower ADC (=higher cellularity) often represent small subpopulations of increasingly dedifferentiated cells. It may be speculated that when these subpopulations grow larger, the skewness and kurtosis of the histogram rise and the risk of lymph node metastasis increases.

The histological grading is an important prognostic factor (19) which may initiate sooner or more aggressive treatment for the individual patient. In the brain, texture features were also found to correlate with the dedifferentiation of gliomas (20). Furthermore, we found that the entropy exhibited near-significant correlation with the histological grade. This is especially interesting since this feature was identified as a surrogate marker in other studies: Kierans et al. have found that malignant adnexal masses have lower entropy than benign ones (5), and Wibmer et al. were able to demonstrate a correlation between the Gleason grade in prostate carcinoma and entropy (6). However, in the latter study the entropy was computed from the GLCM and not from the ADC map; therefore, the results may not be directly comparable. In a murine osteosarcoma model, entropy correlates with apoptosis and cell death (21), meaning that entropy conveys meaningful underlying biological information not only across different entities of cancer but also across different species.

Unexpectedly, texture analysis did not allow for differentiation of the histological tumor type. Given the relatively small sample size used in this pilot study, it would be premature to draw any conclusion from this negative finding; however, it is possible that ultimately other imaging markers may be superior to texture features for prediction of the histological type (such as perfusion parameters obtained by dynamic contrast-enhanced MRI (7) or intravoxel incoherent motion imaging (22)). Although the dominant factors for patient management are FIGO-stage, histological differentiation and nodal status, the histological subtype is also an independent predictor for outcome and treatment response (23). Furthermore, there was no significant correlation with the FIGO stage. This suggests that in the underlying cohort, the tumor texture characteristics were not dependent on the tumor size. It does, however, not mean that there is no tumor heterogeneity but rather that in this small cohort, tumor heterogeneity was not significantly different in different tumor sizes. It has been previously reported that tumor heterogeneity in FDG uptake is correlated with tumor size and cervical stromal invasion (24). One further reason for this discrepancy might be the differences in the used biomarker. FDG measures the metabolic uptake of glucose, whereas DWI is a marker of cell density. From previous studies, a significant correlation between ADC values and tumor differentiation of cervical cancer was reported (25). As the parametrial invasion limits the success of a surgical treatment, the latter distinction is of great clinical importance. Ultimately, like the reading of a radiologist, texture analysis should encompass all sequences. However, as long as their use is still in a preliminary experimental stage, it is important to examine them separately and thoroughly.

Our study has several limitations which need to be stated. First, as a pilot study our cohort was rather small. As already broached above, this could have obscured possible correlations. Second, we only performed texture analysis on a single slice which may not capture the most representative portion of the tumor. Unfortunately, this was a technical necessity because DWI is usually not acquired with isotropic voxel size, and 3D texture analysis would have required interpolation between the single slices, which would have influenced the texture features in an unpredictable way. Therefore, we think working with the original data is superior to analyzing data with an artificially introduced confounder. Third, we only analyzed the ADC maps and not the original image data. Our reason for this is that the choice of the b-value has been shown to confound texture analysis on DW source images, but the features seem more resilient on the computed ADC maps (26).

In conclusion, we have identified texture features which may potentially predict histological tumor differentiation and nodal cancer stage. Further research will be necessary to verify our preliminary findings in a larger cohort.
